# Midwifery educators’ knowledge of antenatal exercises in selected Nigerian midwifery schools

**DOI:** 10.4102/curationis.v47i1.2495

**Published:** 2024-08-16

**Authors:** Mary M. Ojong-Alasia, Seepaneng S. Moloko-Phiri, Molekodi J. Matsipane, Ushotanefe Useh

**Affiliations:** 1 School of Nursing, Faculty of Health Sciences, North-West University, Mahikeng, South Africa; 2 Department of Lifestyle Diseases, Faculty of Health Sciences, North-West University, Mahikeng, South Africa

**Keywords:** antenatal exercises, demonstration, exercise guidelines, knowledge, midwifery

## Abstract

**Background:**

Exercise during pregnancy is beneficial to both the pregnant woman and the foetus. Midwifery educators play a crucial role in ensuring that midwifery students receive the knowledge and training needed to demonstrate antenatal exercises. To ensure that their students understand and deliver adequate antenatal care, midwifery educators should be highly knowledgeable in pregnancy-related exercises.

**Objectives:**

The study was conducted to determine the knowledge of midwifery educators about antenatal exercise.

**Method:**

A descriptive cross-sectional study was conducted of the knowledge about antenatal exercises by midwifery educators. A purposive total population of 54 midwifery educators from three midwifery schools in Cross River State, Nigeria, was included in the study. Questionnaires were used for data collection, and Statistical Package for Social Sciences (SPSS) version 27 was used for data analysis. Ethical issues and rigour were maintained.

**Results:**

The study revealed that antenatal exercises are included in the midwifery curriculum and exercise demonstration were mainly done by midwifery educators and clinical instructors. The majority (*n* = 34, 66.7%) of the respondents were knowledgeable about World Health Organization (WHO) guidelines for exercise during pregnancy and had an average knowledge of the ideal antenatal exercises.

**Conclusion:**

Midwifery educators have average knowledge of the ideal antenatal exercises, which prompts the development of an exercise programme to guide midwifery training and practice. Midwifery educators should collaborate with exercise specialists to teach and demonstrate antenatal exercises.

**Contribution:**

The study highlighted the need for midwifery educators to obtain more information on antenatal exercises to adequately prepare midwifery students for evidence-based exercise care for pregnant women.

## Introduction

Antenatal exercise improves maternal and foetal health and prepares pregnant women for a positive birth experience. Research has disclosed that regular exercise confers significant benefits for the health and wellness of both the mother and the foetus (Gregg & Ferguson 2017:742). The benefits of exercise during pregnancy for mothers include decreased moodiness, reduced risk of gestational diabetes, pregnancy-induced hypertension, improved respiratory function and reduced infant weight among others (Okafor & Goon 2021:787). The authors also opined that increased neurobehavioral development and higher stress tolerance are the advantages of maternal exercise for the foetus.

Midwifery educators play a crucial role in ensuring that midwifery education prepares highly qualified and competent professionals capable of providing an effective response to the various needs of patients and their families (Martins et al. 2018:9). To ensure that student midwives are adequately prepared to deliver safe and appropriate antenatal exercise routines to women, a midwifery education programme must involve midwifery educators with the necessary abilities in theory and practice (International Confederation of Midwives [ICM] 2021:5) including antenatal exercises.

Studies have revealed that quality midwifery education is a key driver for improved quality of health services, which includes antenatal exercises. Education, research and evidence-based practice are interrelated and interdependent; together, they promote lifelong learning and advance the knowledge base of the midwifery workforce to ensure full competency in the administration of best practices in order to guarantee future health gains. Midwifery services must be strengthened by ensuring good educational standards and translating cutting-edge research evidence into people-centred practice (WHO 2017:47).

Midwifery education in Nigeria is categorised into basic and post-basic programmes at the fundamental levels. Midwifery is also offered at the masters and PhD levels. The basic programme is a 3-year course; each year has two semesters with an examination at the end of semester. The final examination is written at the end of the semester in the third year. Successful candidates are allowed to apply for registration and licensing. The basic midwifery programme is for school leavers who must obtain at least five credits including English language, mathematics, physics, biology and chemistry. There are midwifery schools that train mainly community midwifery, which is a 2-year, 4 semester programme, to train students on practical community midwifery skills and give them the knowledge and clinical experience to be licenced community midwives (LCM). The admission requirements for community midwifery are at least four credits including English language and biology, and two science subjects such as physics and chemistry obtained from the West African Examination Council (WAEC), National Examination Council (NECO)/National Business and Technical Examination Board (NABTEB). The community midwife provides basic healthcare during and after pregnancy and childbirth (NMCN 2016a:54, 2016b:34). The post-basic midwifery programme is for 18 months and is for registered nurses only (NMCN 2012:35).

The gap identified in the location for the study was associated with women often presenting at the clinic with ailments associated with pregnancy which were probably indications of inadequate or inappropriate exercises. The types of exercises that clinical midwives utilised for antenatal women were often singing, clapping, and dancing irrespective of the stage of pregnancy. A perusal of the curriculum of midwifery training also showed that there was minimal information on antenatal exercises. The three study areas operate the same curriculum of training developed by the Nursing and Midwifery Council of Nigeria (NMCN) which is the regulatory body for nursing and midwifery training in Nigeria. It was therefore important to assess the knowledge of midwifery educators regarding exercise for pregnant women because they are responsible for the training of midwifery practitioners.

The essence of the study was to bring about enhanced knowledge and change in practice through the development of an exercise programme. Kurt Lewin (1951) change theory was adopted for the study. This theory identified three stages through which change agents must proceed before change becomes part of a system. The stages include (1) the unfreezing stage, (2) change or moving stage and (3) the refreezing stage. The implication for this study, for example, is that midwives have been observed to practice ineffectively in relation to the demonstration of antenatal exercises in Cross River State. The observation called for a change in practice from the current state to a new and standardised mode of practice among midwives with regards to antenatal exercises. Lewin’s second step in the process of change involves changing or moving the current system of practice of demonstration of antenatal exercise to a new level of stability while the final stage, refreezing, is the point of ensuring that the change is made permanent in the organisation. The refreeze step needs to take place after the change has been implemented in order for it to ‘stick’ over time.

Midwifery educators should be knowledgeable of the appropriate exercises during pregnancy while training midwifery students to deliver safe exercise practices to pregnant women. It is observed that many pregnant women continue to present at the clinics with pregnancy-related problems such as lower abdominal and waist pain and pedal oedema, which results from inactivity of the pregnant woman. The common complaints associated with pregnancy such as low back and pelvic pain that many pregnant women in Nigeria complain about are associated with inactivity and related to clinical midwives’ inadequate information about appropriate exercise for pregnant women. The study sought to determine the knowledge of midwifery educators regarding antenatal exercises using a descriptive cross-sectional survey.

## Research methods and design

A descriptive quantitative study was conducted to determine the knowledge of midwifery educators regarding antenatal exercises.

### Setting

The context for this study was Cross River State, Nigeria. Cross River State was selected as the setting of the study because it is one of the states that has midwifery educators and midwifery practitioners, and where midwifery education is carried out according to the prescriptions of the NMCN. It was also observed by the researcher that during antenatal care, clinical midwives engaged these women mainly in exercises such as singing, clapping and dancing, which may not have an adequate impact in reducing and/or preventing ailments associated with pregnancy and childbirth because of improper or inadequate inactivity. At the time of this study, there were three state-owned schools of midwifery; two basic schools and one post basic school.

### Study design

The study was a descriptive cross-sectional survey design, which enabled the researchers to identify key issues and variables relating to the knowledge of midwifery educators regarding antenatal exercises. Cross-sectional surveys are used frequently in nursing research to gather information from respondents to provide good quality data about a problem (O’Connor 2022:1). This study tracks the World Health Organization’s (WHO) new Global Action Plan on Physical Activity 2018–2030 which requests countries to assist people in increasing their levels of physical activity as part of people’s (including pregnant women) everyday lives to promote health, and prevent and manage non-communicable diseases (WHO 2022:n.p.). This study involved midwifery educators from three schools of midwifery in Cross River State Nigeria. Cross-sectional surveys can be considered a snapshot that gives a picture of what the researcher wants to study (Connelly 2016:369). Information from the midwifery educators was obtained during the distribution and retrieval of the questionnaire with no further contact with the respondents.

### Population and sampling strategy

Population refers to a set of similar items that the researcher is interested in to produce information (Dornala and Dornala 2018:144). Martin and Law (2021:438) defined population as the total number of people living in a defined geographical area from which a sample is drawn to conduct a survey. The population for this study consisted of all 54 midwifery educators teaching in the three midwifery schools in Cross River State, Nigeria. The population of midwifery educators in the individual schools was 22 in School **A**, 17 in School **B**, and 15 in School **C**, who all responded in the study.

A purposive non-probability sampling approach was used to select the respondents. Purposive sampling involves the conscious selection of certain subjects to include in a study (Gray, Grove & Sutherland 2017:1081). Midwifery educators are specialists in their field and significant informers regarding antenatal exercises. The total population was used. The use of total population is a procedure where the researcher decides to involve the entire population that has a particular set of characteristics (Ashley 2018:2). The choice of total population was because of the small population size and the uniqueness of the functions of the respondents in relation to the teaching and demonstration of antenatal exercises. The implementation of selection approach of respondents assisted the researchers to give a complete representation of the problem under review and reduced the risk of any missing information. Furthermore, to ensure the population of 54 midwifery educators generated significant results, without a tendency to commit a type two error, statistical power calculation was conducted. Statistical power analyses are used to determine the required sample size to identify the effect of interest with a desired level of statistical power (i.e. the probability to reject an incorrect null hypothesis). Also, statistical power analyses can be used to determine the achieved power of a test, given an effect and a particular sample size (Jobst, Badder & Moshagen 2023:207). Determining the appropriate sample size is an important component of a well-designed research study (Staffa & Zurakowski 2020:1173). A high statistical power of 0.96 was identified for this study, which means the test results of this study are significant.

The population used at the time of the study was the actual number of midwifery educators in the three state-owned schools. There has been a huge loss of staff from the service of the government of Cross River State with no replacement as the government placed a ban on employment. The population size was a limitation for this study; therefore, power calculation was used to ascertain the suitability of the population size to generate significant results. A study with a power of 80% means the study has an 80% chance of the results being significant. A high (90%) statistical power means the test results are most likely significant, and a low (less than 80%) statistical power means the test results are questionable. While a high power in a study indicates a large chance of a test detecting a true effect, low power means that the test only has a small chance of detecting a realistic effect size (Reito, Raittio & Helminen 2020:e0079). Four main factors can affect the power of any test of statistical significance, namely the effect size, the level of significance, the statistical power, and the sample size (Staffa & Zurakowski 2020:1173). The factors are elaborated below.

*The effect size:* Effect size is a measure of the degree to which the null hypothesis is false. Effect size measures are used to quantify treatment effects or associations between variables (Schober, Bossers & Schwarte 2018:1068). It is essential to recognise that power can be deemed adequate with a smaller sample if the effect size is large.

*The level of significance:* The significance level, also known as alpha or α, is a measurement that specifies the amount of evidence that must be shown in the sample before the rejection of the null hypothesis and to declare the effect statistically significant. The significance level is the probability of rejecting the null hypothesis when it is true. A significance level of 0.05 represents a 5% chance of deciding that a difference exists when there is none (Edward & Thomas 2018:691).

The *statistical power* is the probability of rejecting a null hypothesis when it is false (Walmsley & Brown 2017:n.p.). The power of a test to detect a correct alternative hypothesis is the pre-study likelihood that the test will reject the hypothesis; for instance, the probability that *P* will not exceed a pre-specified cut-off, such as 0.05 (Greenland et al. 2016:346).

The *sample size* refers to the number of midwives selected to participate in the study (Polit & Beck 2017:1045). In addition, sample size is an important factor in power analysis. The calculation of each of these factors can only be done in the presence of the other three. Gignac and Szodora (2016:74) give approximate guidelines for estimating effect sizes.

In determining the statistical power of this study, the estimate was that effect size would be medium with a value of 0.5, as shown in Table 1, because of the small population. Level of significance set was thus at 0.05 for a sample size of 54 midwifery educators with the standard error (desired effect size) of 0.5. Therefore, the estimated effect size was calculated at approximate medium level by dividing *d* = *0.5*√/54, *d* = 3.67, and the statistical power was derived from checking the function of *d* = 3.7. Using the level of significance at 0.05 from the power table, the power was approximated as 0.96. The power of 0.96 was a high statistical power, which means the test results of this study are significant.

**TABLE 1:** Estimating effect sizes without knowing the parameters.

Effect size	D	Percent overlap
Small	0.20	85
Medium	0.50	67
Large	0.80	53

*Source*: Gignac, G.E. & Szodora, E., 2016, ‘Effect size guidelines for individual differences researches’, *Personality and Individual Differences* 102(2016), 74–78. https://doi.org/10.1016.paid.06.019

### Data collection

Data were collected using a 14-item self-designed questionnaire on midwifery educators’ knowledge of antenatal exercises. The questionnaire was divided into two sections: section A (demographic characteristics of the respondents’) and section: B (questions to assess respondents’ knowledge of antenatal exercises). A total of 54 questionnaires were distributed directly to the midwifery educators in each school and 51 questionnaires were retrieved same day, which is a 94.4% return rate.

### Validity

Date collection tools used by the researchers were face, and content validity. Face validity is the extent to which a measuring instrument appraises what it purports to measure (Polit & 2017:728). The statistician and peer reviewers assessed the questionnaire and determined at face value that the questionnaire reflected the view of the study. Content validity is the decision made by subject professionals or the application of systematic procedures to examine the test items for appropriateness of the proposed concept (Coe et al. 2017:47). The quantitative verification of validity was through a confirmatory factor analysis (CFA) resulting from an exploratory factor analysis (EFA) which established that the items in the questionnaire were suitable for analysis. Subsequently, an EFA was also used to explore the possibility of establishing the factors describing the midwifery educator’s knowledge and practice of antenatal exercise programme in Cross River State of Nigeria. Exploratory factor analysis is a statistical method used to reveal the basic structure of a tool with large set of variables. Exploratory factor analysis is also a procedure within factor analysis that aims at detecting the essential relationships between measured variables (Warne & Larsen 2014:106). It is also used when the researcher has no previous hypothesis about factors or patterns of measured variables. A CFA was performed on the EFA results to validate these results. Confirmatory factor analysis allows the researcher to test if a relationship between the observed variables and their underlying hidden factors exists (Ruscio & Roche 2012:283). The EFA/CFA are suitable for analysis as the questionnaire for data collection has multivariate items.

### Reliability

Reliability refers to truthfulness in the data obtained and the degree to which any measuring tool controls random error (Ahmed & Ishtiaq 2021:2401). Cronbach’s alpha was used to test the reliability of the items under each construct. In general, Cronbach’s alpha tests have a score range of 0–1. If a score of 0.70 and above is consistently obtained for a particular construct it means that the item is reliable for data collection and to offer significant results (Coe et al. 2017:54). The reliability test for the data collection instrument was 0.9 which ensured that significant results were obtained to meet the aim of the study.

The Cronbach’s alpha statistics was used to determine the reliability of the instrument. Cronbach’s alpha measures the degree to which the items in an instrument are simultaneous. If there is a consistent score of 0.70 and above obtained for a particular construct, then the instrument is reliable and suitable for data collection for a study (Coe et al. 2017:54). Test of reliability was conducted through a test-retest approach, aimed at establishing the correlation of two values obtained within a short period between the tests. Ten midwifery educators from another state in the South-South geopolitical zone, where the study was not conducted were administered the questionnaire and thereafter the researchers evaluated the responses. The 10 midwifery educators from the neighbouring state in the same political zone have similar characteristics as they were all trained under similar midwifery educational systems and adopted the same curriculum from the NMCN. The 10 midwifery educators were only used to test the suitability and/or reliability of the instrument for data collection. The same process was repeated 1 week later when new copies of the same questionnaire were administered to the same group of midwifery educators. The scores were 0.70 and 0.80, respectively which meant the instrument was reliable for data collection.

The gap identified was in the inadequate practice of exercise by clinical midwives and to determine if this inappropriateness was associated with the knowledge of exercises by the midwifery educators who trained the clinicians and to establish the adequacy of the training curriculum with regards to content for exercises during pregnancy.

### Data analysis

The International Business Machine (IBM), headquartered in Armonk, New York, USA, Statistical Package for the Social Sciences (SPSS) Version 27.0 was used to analyse the data 154 that were exported from the Excel sheet. The results of the statistical analysis of descriptive 155 statistics were displayed in tables, pie charts and bar charts.

### Ethical considerations

Ethical clearances to conduct the study were obtained from the Health Research Ethics Committees of North-West University, South Africa (HREC number: NWU-00475-19-A1) and the Cross River State of Nigeria Ministry of Health, Health Research Ethical Committee (reference number CRSMOH/RP/REC/2021/149). Approval was also granted by the principals of the three midwifery schools on presentation of the ethical clearance from the Ministry of Health. Respondents were given adequate information about the research through information pamphlets which enabled them to consent or decline participation. Both verbal and written consent was obtained from all the respondents without coercion before their participation in the study. Respondents were informed that the data obtained were for research purposes only and no unauthorised persons will have access to the information. For anonymity and confidentiality, respondents were not required to write their names; rather, codes were assigned to their responses and information stored in password-protected computers and retrieved questionnaires were kept in a locked file cabinet.

## Results

### The inclusion of antenatal exercises in the curriculum of midwifery training

**FIGURE 1:**
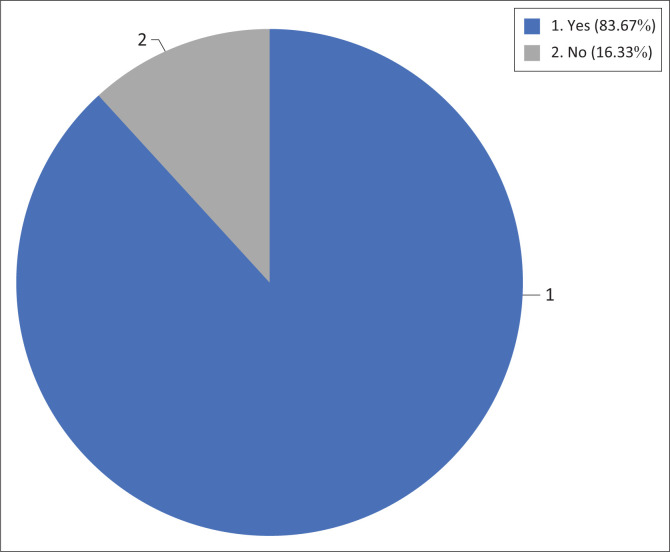
Are antenatal exercises included in the curriculum of midwifery training?

**FIGURE 2:**
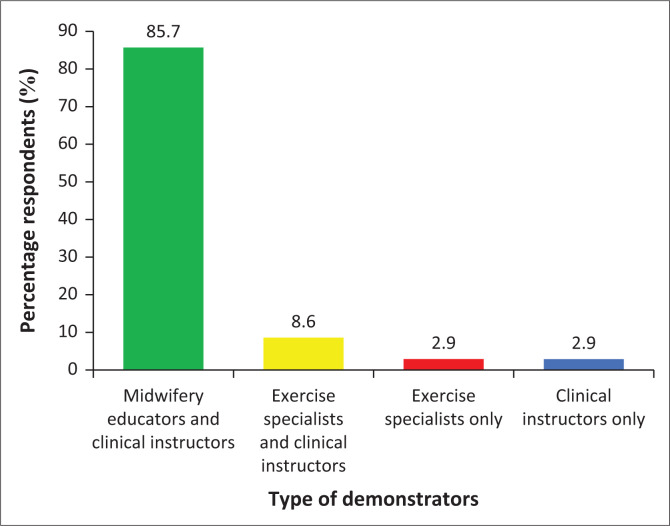
Personnel who conduct practical demonstration of antenatal exercises to the learners.

The study determined if antenatal exercises were included in the midwifery curriculum. The result shows that 43 (83.67%) respondents indicated that antenatal exercises were included in the curriculum of midwifery training, while 8 (16.33%) specified there was no content in the midwifery curriculum (Figure 1). The results revealed the personnel responsible for the demonstration of antenatal exercises to the midwifery students as depicted in Figure 2.

### Personnel who conduct practical demonstration of antenatal exercises to learners

**FIGURE 3:**
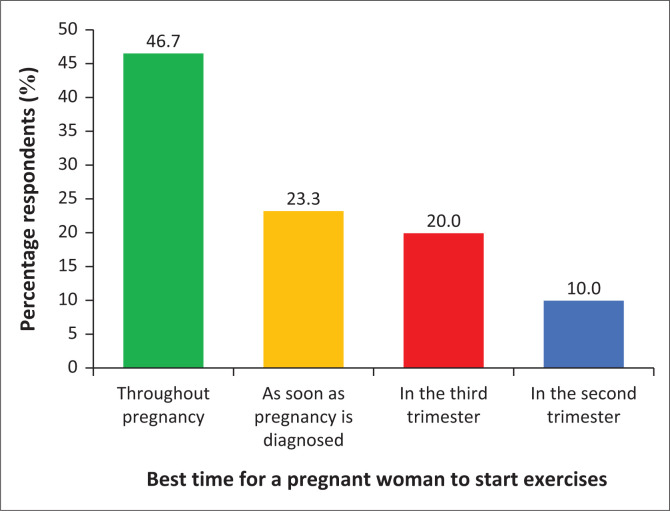
The best time for a pregnant woman to start exercise.

Figure 2 depicts the responses on the personnel who conduct practical demonstration of antenatal exercises to learners. Results revealed that majority *n* = 44 (85.7%) of respondents indicated that midwifery educators and clinical instructors conduct practical demonstration of antenatal exercises, *n* = 5 (8.6%) respondents indicated midwifery educators, exercise specialists and clinical instructors conducted the demonstration of antenatal exercises to learners. The results also highlighted the best time for the pregnant woman to start exercise as presented in Figure 3.

### The best time for a pregnant woman to start exercises

Figure 3 depicts the responses on the best time to start exercise during pregnancy. The results also revealed that majority *n* = 24 (46.7%) of the respondents specified that it is best for the pregnant woman to exercise throughout pregnancy, while *n* = 12 (23.3%) respondents suggested that antenatal exercise should begin as soon as pregnancy is diagnosed, meanwhile *n* = 10 (20.0%) respondents indicated that antenatal exercise should begin in the third trimester, and *n* = 5 (10.0%) respondents specified that pregnant women should start exercise in the second trimester. Respondents also provided information on WHO-recommended guidelines for exercises during pregnancy.

### The World Health Organization recommended exercise guidelines for pregnant women

**FIGURE 4:**
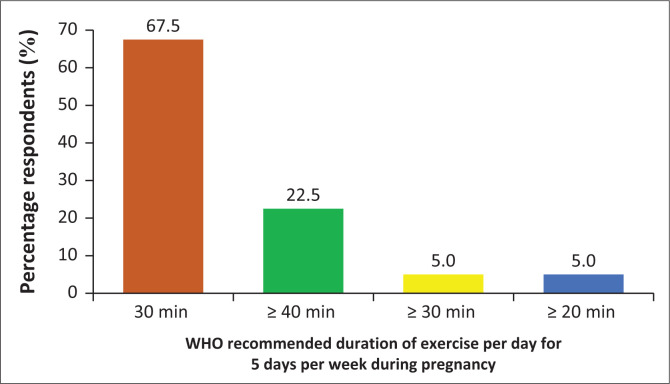
World Health Organization (WHO) guidelines for exercise during pregnancy (moderate intensity cardiorespiratory exercises).

Responses about WHO’s recommended guidelines for exercises during pregnancy revealed that majority *n* = 34 (66.7%) of the midwifery educators submitted that 201 pregnant women should engage in moderate intensity cardiopulmonary exercise training for 30 min/day for 5 days a week, for a total of 150 min/week (Figure 4). Information on the ideal exercises for each trimester was provided by the respondents as highlighted in the subsequent paragraphs.

### Knowledge of ideal exercises across the three trimesters

Table 2 presents the results about the knowledge of midwifery educators regarding ideal exercises during the three trimesters. Twenty-one exercises were included for the identification of the ideal exercises for each trimester. The responses about respondents’ knowledge of ideal exercises during the three trimesters are based on results for each individual exercise across the three trimesters.

**TABLE 2:** Responses regarding suitable exercises across the three trimesters.

Exercises	First trimester				Second trimester				Third trimester			
	Agree		Disagree		Agree		Disagree		Agree		Disagree	
	*n*	%	*n*	%	*n*	%	*n*	%	*n*	%	*n*	%
Brisk walking	26	51	25†	49†	-	-	-	-	-	-	-	-
Dancing (moderate intensity)	26	51	25†	49†	46†	90†	5	9	46	90	5†	10†
Flexing	22†	443†	29	57	-	-	-	-	-	-	-	-
Foot and ankle exercise	-	-	-	-	-	-	-	-	-	-	-	-
Household chores	-	-	-	-	-	-	-	-	49†	96†	2	4
Jogging	20†	39†	31	61	-	-	-	-	-	-	-	-
Kegel’s exercise	28†	55†	23	45	-	-	-	-	36†	70†	15	30
Lifting dumb bells	18†	35†	33	65	17†	33†	34	67	11†	22†	40	78
Pelvic tilts	21	41	30†	59†	24†	47†	27	53	28†	55†	23	45
Rope jumping or skipping	16†	31†	35	69	13	25	38†	74†	29	57	22†	43†
Running	11†	22†	40	78	17†	33†	34	67	-	-	-	-
Rowing	26†	51†	25	49	-	-	-	-	25†	48†	26	52
Sit-up or press-ups	20†	39†	31	61	-	-	-	-	-	-	-	-
Slow walking or gentle walks	-	-	-	-	-	-	-	-	-	-	-	-
Squatting	28†	55†	23	45	26†	51†	25	49	32†	63†	19	37
Stability ball exercise	35†	69†	16	31	39†	77†	12	23	37†	72†	14	28
Stair climbing	-	-	-		-	-	-	-	-	-	-	-
Stationary cycling	34†	67†	17	33	-	-	-	-	29†	57†	22	43
Stretching	-	-	-	-	-	-	-	-	28†	55†	23	45
Swimming	38†	74†	13	26	-	-	-	-	35†	69†	16	31
Yoga	25†	49†	26	51	35†	69†	16	31	34†	67†	17	33

† , some of the appropriate exercises indicated by the respondents across the trimesters.

### Ideal exercises during the first trimester (0 – week 12)

The results revealed that the ideal exercises identified by the respondents for the first trimester included flexing *n* = 22 (44.3.%), jogging *n* = 20 (39.0%), Kegel’s pelvic floor exercise *n* = 28 (55.0%); lifting of thumb-bells *n* = 18 (35.0%), rope skipping *n* = 16 (31.0%), running *n* = 11 (22.0%), rowing *n* = 26 (51.0%), sit-ups/press-ups *n* = 20 (39.0%), squatting *n* = 28 (55.0%), stability ball exercise *n* = 35 (69.0%), stationary cycling *n* = 34 (67.0%), swimming *n* = 38 (74.0%) and yoga *n* = 25 (49.0%) The respondents also identified exercises considered unsuitable during the first trimester to include brisk walk *n* = 25 (49.0%) dancing *n* = 25 (49.0%) and pelvic tilts *n* = 30 (59.0%). The ideal exercises for the second trimester were also signified by the respondents.

### Ideal exercises for the second trimester (week 13 – week 28)

The respondents identified the following exercises as ideal during the second trimester: moderate intensity dancing *n* = 46 (90.0%), lifting dumb-bells *n* = 17 (33.0%), pelvic tilts *n* = 24 (47.0%), running *n* = 17 (33.0%), squatting *n* = 26 (51.0%), stability ball exercise *n* = 39 (76.0%). and yoga *n* = 35 (69.0%). The respondents disagreed that rope skipping was suitable during the second trimester. The respondents also identified the ideal exercises for the third trimester.

### Ideal exercises for the third trimester (week 28 – week 40+)

Exercises identified by the respondents as appropriate during the third trimester included: household chores *n* = 49 (96.0%), Kegel’s exercise *n* = 36 (70.0%), lifting dumb-bells *n* = 11 (22.0%), pelvic tilts *n* = 28 (55.0%), rowing *n* = 25 (49.0%), squatting *n* = 32 (63.0%), stability ball exercise *n* = 37 (72.0%), stationary cycling *n* = 29 (57.0%), stretching *n* = 28 343 (55.0%), swimming *n* = 35 (69.0%) and yoga *n* = 34 (67.0%). The respondents disagreed that in the third trimester moderate intensity dancing *n* = 5 (10.0%) and rope skipping *n* = 22 (43.0%) were ideal at this period. The results revealed the overall knowledge of respondents regarding the ideal antenatal exercises across the trimesters.

### Overall knowledge of ideal antenatal exercises

**FIGURE 5:**
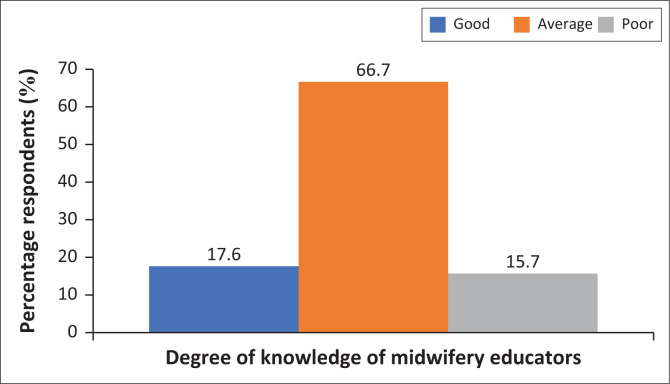
Overall knowledge of midwifery educators regarding suitable antenatal exercises.

Figure 5 shows the overall results of the respondents’ knowledge of ideal antenatal exercises. To reveal the respondents’ overall knowledge of the ideal antenatal exercises, the data were aggregated, graded, computed in percentages, and the results were displayed in a bar chart. Each correct choice was allotted a score of 1, whereas an incorrect response was assigned a score of 0. Minimum and maximum scores ranged from 21.00% to 81.00%; good knowledge was defined as a score over 70.00%, average knowledge as a score between 50.00% and 69.00%, and low knowledge as a score between 0.00% and 49.99%. As depicted in Figure 5, 17.6% of the respondents had a good understanding of appropriate antenatal exercises, 66.7% had moderate knowledge, and 15.7% had poor knowledge of antenatal exercises. The ability to identify accurate and erroneous choices for the final grade of knowledge as good, average or poor was confirmed from the reviewed literature.

## Discussion

This study sought to document the Nigerian midwifery educators’ knowledge of exercises and their suitability across the three trimesters in selected midwifery schools. Overall, the midwifery educators indicated that antenatal exercises were included in the midwifery curriculum. The results are supported by United Kingdom Physical Activity Fact Sheet (UKPAF 2018:1), which recommended that physical activity education should be part of the midwifery curriculum. However, some respondents specified that there was no content for antenatal exercises in the midwifery curriculum. The results may mean that the curriculum does not have content for antenatal exercises. This outcome is comparable to research conducted in Kenya where antenatal exercise materials were not included in the diploma nurse programme (Vurigwa, Edwin & Donald 2020:31). Similarly, the results of a review of the WHO 4-year integrated nursing and midwifery competency-based pattern curriculum for African region had no content for exercises for pregnant women (WHO 2016:102). The inference from these findings is that the curriculum for midwifery training maybe inadequate which could have a negative impact on midwifery students’ acquisition of knowledge and skills related to antenatal exercises. Oh and Rozycki (2017:1) reported that an inadequate curriculum content can lead to a deficiency in learners’ professional development. A review of the midwifery curriculum to reflect content for exercises for pregnant women is important to enhance midwifery training and practice. All the midwifery educators indicated that they were actively involved in teaching antenatal exercises. The fact that all midwifery educators in the three schools are involved in teaching and demonstrating antenatal exercises is because of the lack of exercise specialists in the country. Nigeria with a population of over 200 million, and only has 2450 physiotherapy exercise professionals, or 0.13 physiotherapists per 100 000 persons (Physiotherapy Society of Nigeria [PSN] 2020:1). The findings revealed that antenatal exercises are currently taught by midwifery educators; however, there is a need for more result-oriented teaching through collaboration with exercise professionals. The majority of the respondents specified that the demonstration of antenatal exercises is majorly the responsibility of midwifery educators and clinical midwives. The result is in line with the report from a study conducted in Australia which revealed that clinical learning and development of competence of midwifery learners are the responsibility of educators and clinical preceptors (Griffiths, Creedy & Carter 2021:e14; Griffiths et al. 2020:240). The results of a cross-sectional study in Tanzania established that it is the responsibility of the midwifery educators and clinical instructors to assist nursing and midwifery students acquire clinical skills through the demonstration of midwifery procedures (Gemuhay 2019:4). However, some of the respondents reported that midwifery educators, exercise specialists and clinical instructors conduct the practical demonstrations of antenatal exercises. This is only possible when there is collaboration between exercise specialists and midwives. Ojukwu et al. (2018:3) state that the teaching and demonstration of exercises is primarily the role of trained physiotherapists, knowledgeable in biomechanics, kinesiology and exercise therapy. The acute shortage of exercise specialists and/or physiotherapists in Nigeria is a key gap for meeting the exercise needs of pregnant women thereby, making it imperative for midwives such as midwifery educators to fill-in the gap during midwifery training. At this point, collaboration between midwifery educators and exercise specialists is necessary to provide safe exercise education and skills. The World Health Professions Alliance (WHPA) asserts that inter-professional collaborative practice provides all-inclusive, harmonised and safe healthcare responsive to the needs of the population, among other benefits (WHPA 2019:1). The inference from these submissions is that the demonstration of antenatal exercises should be the collaborative effort of midwifery educators, clinical instructors and exercise specialists. Information on the best time for a pregnant woman to begin exercising varied widely among respondents. The majority of respondents also suggested that pregnant women should exercise all through the pregnancy. The opinion of the majority of the respondents is in line with the recommendation of the WHO that all expectant women should engage in regular physical activity throughout the pregnancy. Meanwhile, some of the respondents submitted that it was best for the pregnant woman to start exercising as soon as pregnancy is diagnosed. This view is supported by some exercise experts, who recommend that women who were active before becoming pregnant should keep up their usual exercise regimen and start exercising in the first trimester (Carey 2020:n.p.; Huizen 2021:n.p). Other researchers suggested that pregnant women who routinely engaged in vigorous-intensity aerobic activity or other forms of physical activity before to becoming pregnant can continue to do so (Bull et al. 2020:1456). Some respondents suggested that it was best for the pregnant woman to start exercising in the second trimester. This finding agrees with the position of the American College of Obstetricians and Gynaecologists (ACOG 2020:135) which advised that women who were sedentary before becoming pregnant should start regular exercise in the second trimester. Furthermore, some respondents indicated that the third trimester was the best time to start antenatal exercise. The result aligns with the findings from a cross-sectional survey among pregnant women in Nigeria, which revealed that most pregnant women engaged in antenatal care late and frequently began exercising in the third trimester (Ojukwu et al. 2018:3). The varied positions of the respondents on the best time to start antenatal exercises are supported in literature which implies that there is no fixed time that a pregnant women could start exercise. Therefore, midwives should adopt a person-centred approach when organising exercise interventions to address the specific needs of the pregnant woman.

An understood concept that retards the goal to reduce maternal mortality and increase universal access to reproductive health including exercise participation is disrespect and abuse (D&A) during childbirth. Educating healthcare providers and women about their responsibilities and rights will enhance provision and utilisation of quality maternal health services (Amole et al. 2019:21). Scientists recognise that large inter-individual differences in adaptation to pregnancy exist (Forczek & Staszkiewicz 2021:114), and every woman solves this problem on her own. Women react to pregnancy differently; there should be no ‘right’ moment for every woman to start exercising; rather, the pregnant woman should begin exercise whenever it is safe and comfortable. Santos-Rochas & Szumilewicz (2019:183) suggest that health-related fitness exercise assessments should take into account the body adaptations and the pregnancy-related symptoms of each stage of pregnancy in order to provide safe and effective exercise. The majority of the respondents were reported to be knowledgeable about the WHO recommended guidelines for exercises during pregnancy which states that a pregnant woman should engage in moderate intensity cardiopulmonary exercise training for 30 min/day for 5 days a week, for a total of 150 min/week. The result tallies with the WHO recommendation of moderate-intensity exercise at 30 min/day for 5 days a week, at 150 min per week (WHO 2018:1). The results imply that the respondents teach students the appropriate exercise guidelines during pregnancy. However, the finding is contrary to the report that midwives in Africa, including Nigeria and South Africa, were perceived to lack knowledge on the safety of exercises, weight gain during pregnancy and types of exercises that can be done by the pregnant women. This inadequate knowledge leads to the distrust in midwives and a decrease in a pregnant women’s adherence to physical activity (Koeller 2022). Also, research has shown that nurses have a good attitude in promoting antenatal physical exercises in Kakamega county; however, the low knowledge affects their practice of exercise care (Vurigwa et al. 2020:vi). The results are also dissimilar to the findings in the United Kingdom by Hopkinson et al. (2018:25) and Lieferman et al. (2018:168), whose findings in the United States revealed that midwives have deficient knowledge about physical activity guidelines. The results revealed the knowledge of the respondents regarding the ideal exercise for each trimester. In the first trimester, flexing, jogging, lifting dumbbells weighing 1.5 kg to 2 kg, skipping rope, running, rowing, sit-ups/press-ups, squats, the Kegel exercise, swimming and yoga were identified by respondents as the ideal exercises. The result is supported by Huzien (2021:n.p.) who suggested that these exercises are appropriate at low intensity during the first trimester. Paola, Montpeptit-Huynh and Vopni (2019:22) emphasised that squats enhanced strength and stamina of the gluteal and leg muscles. Millard (2021:1) indicated that sit-ups are safe during the first trimester. However, less than 50% of respondents disagreed that pelvic tilts, moderate-intensity dancing and brisk walking are ideal exercises during the early stages of pregnancy. The suggestions that pelvic tilts, moderate-intensity dancing and brisk walking are unsuitable in the first trimester tally with the advice that low-intensity activities are appropriate during the first few weeks of pregnancy until the woman feels more energised (Paola et al. 2019:4). Pelvic tilt exercise is recommended as appropriate from the fourth month of pregnancy, and specifically from the 33rd week (Tian 2018:n.p.). It could be inferred from the differences in opinion about the ideal exercises during the first trimester that midwifery educators do not have adequate knowledge regarding antenatal exercises. The inadequate knowledge of midwifery educators is supported by Hopkinson et al. (2018:25) and Lieferman et al. (2018:168), who reported that midwives lack adequate knowledge about suitable exercises during the first trimester. Ideal exercises identified by the respondents as the best forms of exercise for the second trimester include moderate intensity dancing, pelvic tilts, lifting dumbbells, running, stability ball exercises, squatting and yoga. The result is supported by Bellefonds (2019:n.p.), who indicated that moderate-intensity dancing brisk walking and running are exercises pregnant women can engage in during the second trimester. Ninety of respondents specified that moderate intensity dancing is an ideal exercise during the second trimester, which may suggest that this is a frequent exercise among pregnant Nigerian women. The results from a cross sectional study conducted among pregnant women at the University of Calabar Teaching Hospital, Nigeria, support dancing as a common exercise during pregnancy, and majority of the pregnant women engaged in dancing as a form of exercise (Umoe et al. 2020:162). Carey (2020:3) advised that yoga stretches the muscles and reduces pregnancy pains, such as lower back pain, and decreases blood pressure while Tian (2018:n.p.) asserted that the pregnant woman could start engaging in pelvic tilt exercise in the second trimester. Soma-Pillay et al. (2016:92) and Nair (2018:n.p.) suggested that antenatal exercises during the second trimester could increase in intensity as most pregnant women are more energetic having recovered from the effects of hyperemesis. More exercises that are safe to engage in the second trimester were also found in the literature reviewed and include brisk walks, flexing, foot and ankle exercises, doing housework, Kegel’s exercises, rowing, squatting, stability ball exercises, climbing stairs and yoga (Holland 2017:1; Sports Medicine Australia 2016:1). Majority of the respondents disagreed that rope skipping was ideal during the second trimester. The result is in harmony with the advice of many doctors who suggest that pregnant women should refrain from jumping exercises, such as skipping ropes, which may increase the risk of health complications during pregnancy (Gynt 2021:n.p.; Pillai 2021:n.p.). In addition, the authors noted that the frequency of jumping and bouncing slackens the ligaments and increases the risk of injury and miscarriage. Millard (2021:1) suggested that exercises in the supine position should be avoided during the second trimester because at this stage the gravid uterus presses on the inferior vena cava; lying flat on the back during the second trimester and beyond might cause a reduction in blood pressure and lead to fainting. The inference from the results implies that the differences in respondents’ views mean midwifery educators have inadequate knowledge about ideal antenatal exercises for the second trimester. The respondents identified exercises appropriate during the third trimester to include: household chores, Kegel’s exercise, lifting dumb-bells, pelvic tilts, rowing, squatting, stability ball exercise, stationary cycling, stretching, swimming and yoga. Many researchers support these results as shown in the discussion. Stability ball exercise was reported to be beneficial for building endurance in the legs and pelvic floor, and to alleviate low back pain in pregnancy (Aftab et al. 2021:256; Piercy et al. 2018:2022). In addition, the authors indicated that flexibility and stretching exercises improve the range and ease of movement around the joints while yoga combines muscle-strengthening, light-intensity aerobic activity and flexibility exercises, among others. Furthermore, the authors suggested that pregnant woman could continue with light exercises, such as gentle walks, ankle, and leg exercises, and breathing exercises during the third trimester. Exercise experts also suggested the encouragement of low to moderate-intensity physical activity during the third trimester. For example, Marcin (2018:n.p.), Paolo et al. (2019:220), and Terreri (2018:n.p.) support this assertion, and pointed that a woman’s energy level reduces in the third trimester; therefore, a reduction in the intensity and duration of exercise is essential. This result is supported in the findings by Daneau et al. (2021:2) who reported that in the third trimester, the pregnant woman may experience instability in the pelvis because of effects of ‘relaxin’, a female reproductive hormone produced by the ovaries and placenta. The role of relaxin is to relax spinal and pelvic ligaments and joints in order to facilitate child birth (Daneau et al. 2021:2). It loosens and relaxes the muscles, joints, and ligaments during pregnancy’ and causes joint instability and reduces agility that may result in falls. Women have relaxin receptors in multiple joints including the hip and knee, and increased relaxin correlates with increased musculo-skeletal injuries (Parker et al. 2022:). Relaxin (RLX) is a hormone primarily secreted by the corpus luteum that results in haemodynamic changes such as loosening the pelvic ligaments during pregnancy (Wang et al. 2021:91). Furthermore, the results revealed that some respondents disagreed that moderate intensity dancing was an ideal exercise during the third trimester. The idea of reducing the intensity, volume and duration of dancing during the third trimester of pregnancy needs to be reviewed. A Nigerian dancer and mother, Korra Obidi, Gravida 2 Para2, all alive, engaged in vigorous belly dancing throughout her pregnancy. She reportedly took part in the ‘So you think you can dance’ audition in California (Nairaland Forum 2019:n.p.). One of the judges at the audition said to her ‘You are a world-changer, a systems-breaker’. The performance of this artiste calls for a review of recommendations of the intensity and volume of dance activities during the third trimester. In addition, some other respondents disagreed that rope skipping is suitable in the third trimester. Most up-to-date exercise guidelines are not trimester specific, Beetham et al. (2019:17) suggest it is important to vary the intensity and duration of exercises at each trimester because of the increasing physiological stresses that occur during pregnancy. In addition, the authors advocate for exercise programmes during pregnancy that should be individualised and conducted with guidance from an exercise professional. The overall results on respondents’ knowledge of ideal exercise across the trimesters revealed that majority of the respondents had average knowledge, while less than 20.0% had good knowledge and 15.7% had poor knowledge of ideal exercises during pregnancy. The inadequate knowledge of midwifery educators regarding ideal exercises during pregnancy is similar to the findings of a survey among medical practitioners in Australia, which revealed that although the majority of the participants agreed that exercise was beneficial to the mother and unborn child, the respondents lacked knowledge pertaining to the type of exercise that the pregnant woman should engage in (Hayman et al. 2017). The results are further supported by findings from Hopkinson et al. (2017:25) and Lieferman et al. (2018:168), which indicated that midwives lacked sufficient information about appropriate exercises for each trimester. The inference from the results is that although majority of the respondents had average knowledge of ideal exercise across the trimesters, there is a need for further training to update information on suitable exercises during pregnancy, with the emphasis on each trimester.

### Limitations

The main drawback of the study was the limited sample size of midwifery instructors. Another difficulty with discussing the results of this study with other comparable studies was the lack of literature on studies among midwifery educators in relation to prenatal activities.

### Implications and recommendations

The implications are that the study results will add to the body of knowledge of midwifery profession as most midwifery curricula do not have content for antenatal exercises. Graduates of the midwifery programme will acquire knowledge and skills regarding antenatal exercises to positively impact on patients’ well-being. The results will form a basis for policy regarding the inclusion of antenatal exercises in the midwifery curriculum. The recommendations arising from this study focus on advocating for physical education classes to be part of the curriculum for midwifery training. Regular workshops, conferences and seminars should be organised on antenatal exercises for midwifery educators to have up-to-date information and enhance practice. Exercise specialists should be deployed to schools of midwifery to collaborate with midwifery educators to teach and demonstrate antenatal exercises. The study should be replicated with a larger population of midwifery educators and in more schools of midwifery in other states to generate more information to improve midwifery education and practice. Additionally, a qualitative research of midwifery educators may produce more detailed information about their understanding of antenatal exercises.

## Conclusion

The results revealed that majority of the respondents indicated that antenatal exercises were included in the midwifery curriculum. The demonstration of antenatal exercises is the joint responsibility of midwifery educators and clinical midwives. Diverse views were expressed about the best time for a pregnant woman to start exercising. Women react to pregnancy differently and there should be no ‘right’ moment for every woman; preferably, the pregnant woman should be encouraged to begin exercising when it is safe and comfortable. It is important for medical screening to be carried out to begin or continue an exercise programme to guarantee a low-risk exercise during pregnancy. Most of the respondents have up-to-date knowledge of the WHO recommendation for exercise during pregnancy, which implies they use the guidelines during teaching and demonstration of antenatal exercises. Various antenatal exercises were recommended across the three trimesters to be performed with different intensities and durations. Overall, the results revealed that most midwifery educators had average knowledge about ideal antenatal exercises.
